# Un-explained visual loss following silicone oil removal: results of the Pan American Collaborative Retina Study (PACORES) Group

**DOI:** 10.1186/s40942-017-0079-6

**Published:** 2017-07-24

**Authors:** Jose A. Roca, Lihteh Wu, Maria Berrocal, Francisco Rodriguez, Arturo Alezzandrini, Gustavo Alvira, Raul Velez-Montoya, Hugo Quiroz-Mercado, J. Fernando Arevalo, Martín Serrano, Luiz H. Lima, Marta Figueroa, Michel Farah, Giovanna Chico

**Affiliations:** 1 Clinica Ricardo Palma, Lima, Peru; 2 Asociados de Macula Vitreo y Retina de Costa Rica, Apdo 144-1225 Plaza Mayor, San José, Costa Rica; 3 University of Puerto Rico, San Juan, Puerto Rico; 4 Fundación Oftalmológica Nacional, Universidad del Rosario, Bogotá, Colombia; 5 OFTALMOS, Catedra de Oftalmologia, Universidad de Buenos Aires, Buenos Aires, Argentina; 6 Centro de Macula, Bogota, Colombia; 7 Asociación Para Evitar la Ceguera, Hospital Luis Sanchez Bulnes, Mexico, DF Mexico; 8 Wilmer Eye Institute, Johns Hopkins University, Baltimore, MD USA; 9 Clinica Oftalmologica Centro Caracas, Arevalo-Coutinho Foundation for Research in Ophthalmology, Caracas, Venezuela; 10 Departamento de Oftalmologia, Instituto da Visão, Universidade Federal de São Paulo, Sao Paulo, Brazil; 11 Departamento de Retina, Hospital Universitario Ramón y Cajal, Madrid, Spain

**Keywords:** Un-explained visual loss, Silicone oil, Vitrectomy, Retinal detachment, Müller cells, Neuronal apoptosis, Potassium

## Abstract

**Purpose:**

To report the incidence and clinical features of patients that experienced un-explained visual loss following silicone oil (SO) removal.

**Methods:**

Multicenter retrospective study of patients that underwent SO removal during 2000–2012. Visual loss of ≥2 lines was considered significant.

**Results:**

A total of 324 eyes of 324 patients underwent SO removal during the study period. Forty two (13%) eyes suffered a significant visual loss following SO removal. Twenty three (7.1%) of these eyes lost vision secondary to known causes. In the remaining 19 (5.9%) eyes, the loss of vision was not explained by any other pathology. Eleven of these 19 patients (57.9%) were male. The mean age of this group was 49.2 ± 16.4 years. Eyes that had an un-explained visual loss had a mean IOP while the eye was filled with SO of 19.6 ± 6.9 mm Hg. The length of time that the eye was filled with SO was 14.8 ± 4.4 months. In comparison, eyes that did not experience visual loss had a mean IOP of 14 ± 7.3 mm Hg (*p* < 0.0002) and a mean tamponade duration of 9.3 ± 10.9 months (*p* < 0.0001).

**Conclusions:**

An un-explained visual loss after SO removal was observed in 5.9% of eyes. Factors associated with this phenomenon included a higher IOP and longer SO tamponade duration.

## Background

Silicone oil (SO) has long been used as a long term intraocular tamponading agent for complex retinal detachments associated with severe proliferative vitreoretinopathy (PVR) [1], giant retinal tears (GRT) [2], tractional retinal detachment in proliferative diabetic retinopathy [3, 4], viral retinitis [5, 6], and trauma with PVR [7, 8]. SO is generally well tolerated. However complications associated with intraocular SO tamponade such as the development of cataract, glaucoma and keratopathy may occur [9–12]. Therefore, in order to lessen these complications, several authors have recommended the extraction of the SO as soon as a stable situation in the retina has been achieved [13–15]. The removal of SO is also typically advocated because removal is associated with improved visual acuity in approximately 30% of patients [13]. On the other hand, visual loss may occur following SO removal. Causes of this visual loss include retinal re-detachment, optic nerve damage due to glaucoma, hypotony, dense vitreous hemorrhage, expulsive hemorrhage, and corneal abnormalities [16]. Over the past decade several groups have reported an un-explained visual loss following SO removal [17–22]. The exact nature of this outcome remains unknown. Our purpose is to report the incidence and clinical features of patients that experienced un-explained visual loss following SO removal.

## Methods

We conducted a multicenter retrospective study of all patients that underwent SO removal from an eye during 2000–2012 at 11 centers in Latin America and Spain. Patients with incomplete records and eyes with retinopathy of prematurity were excluded. SO was removed from the eye and exchanged with BSS in all cases.

Data collected from each patient included the primary diagnosis, best corrected visual acuity before and 1 month, 6 months and 12 months after the removal of SO, SO viscosity, number of surgeries prior to the SO extraction, duration of SO tamponade, IOP before initial vitreoretinal surgery, IOP during SO tamponade and ocular co-morbidities. SD-OCT and FA were performed in eyes that experienced loss of visual acuity with no apparent cause.

At very low values of VA, it is difficult to quantitatively assess the degree of visual loss. However, all of the patients that experienced loss of vision brought it to the attention of their physicians, in fact they were quite upset. Thus for patients with a symptomatic loss of vision and a visual acuity of <20/400, a 20/200 “E” card was held at different distances until the patient was able to see it. This was repeated at least 3 times. Hand motions vision was determined if the 20/200 “E” card could not be seen at 1 foot and yet the patient was able to perceive hand motions. Patients who could perceive only light or who could not perceive light were recorded as such. Hand motions and light perception acuity were assigned logMAR scores that were 0.1unit (or 1 line of acuity) higher than the logMAR score corresponding to the lowest acuity measured with optotypes (1/200 Snellen, 5/200 ETDRS). Given the retrospective nature of the study, this assessment of visual acuity was only performed in patients that were symptomatic and not on all patients with a visual acuity <20/400. In the logMAR scale each line of VA is 0.1 logMAR units so 2 lines would be 0.2 logMAR units. Eyes were determined to have a loss of vision if the BCVA following SO removal was ≥2 lines when compared to the BCVA prior to SO removal.

### Statistical methods

Statistical analysis was performed using the statistical software Stata V10 (StataCorp LP, College Station, TX, USA). Patients’ BCVA were transcribed from their records and converted to a logarithm of the minimal angle of resolution (logMAR) scale for analysis. Count fingers, hand motion, light perception and no light perception were assigned logMAR values of 2, 2.3, 2.6 and 2.9 respectively [23]. Fishers exact test was used to compare categorical data. The odds ratio and the confidence intervals were calculated for these variables. Non parametric ANOVA with Mann–Whitney test was used to analyze continuous variables. A *p* value <0.05 was considered to be statistically significant.

## Results

A total of 324 eyes underwent SO removal during the study period.

Forty two (13%) eyes suffered a significant visual loss following SO removal.

Twenty three (7.1%) of these eyes lost vision secondary to known causes such as retinal re-detachment and proliferative vitreoretinopathy (7 eyes), vitreous hemorrhage secondary to diabetic retinopathy (3 eyes) and glaucoma (13 eyes).

In the remaining nineteen (5.9%) eyes, the loss of vision was not explained by any other pathology. Eleven of these 19 patients (57.9%) were male. The mean age of this group was 49.2 ± 16.4 (range 16–73) years. Systemic co-morbidities included 9 patients with diabetes mellitus and 8 patients with systemic hypertension. In 11 patients 5000 cs SO was used and in 8 eyes 1000 cs SO was used. In all of these cases the retina remained attached during the silicone oil fill and following its removal. SD-OCT and FA were performed in all these cases and did not reveal any causes of acute visual loss such as cystoid macular edema, epiretinal membrane or macular ischemia. Ultrastructural evaluation of the OCT images did not reveal changes in the integrity of the ellipsoid, external limiting membrane and interdigitation zones. In addition, in these eyes with un-explained visual loss following silicone oil removal the IOP remained under 23 mm Hg during the entire post-operative and silicone oil tamponade periods.

Eyes with un-explained visual loss and those without visual loss were compared with regard to several variables including gender, age, systemic co-morbidities (diabetes mellitus, cardiovascular disease, systemic hypertension), indication for initial surgery (retinal detachment with giant retinal tear, retinal detachment with proliferative vitreoretinopathy, diabetic tractional retinal detachment, retinal detachment secondary to viral retinitis etc.), pre-operative macular status, baseline pseudophakia, intraocular pressure during SO fill, length of time of SO tamponade and viscosity of SO. We identified a higher intraocular pressure and a longer length of time of silicone oil tamponade as factors associated with un-explained visual loss. Eyes that lost vision had a mean IOP while the eye was filled with SO of 19.6 ± 6.9 mmHg (range 11–23). None of the eyes had an IOP spike higher than 24 mm Hg during SO tamponade. To assess for IOP fluctuations we calculated the differences in IOP between the maximum and minimum IOPs for each individual eye. We compared the IOP fluctuations during SO tamponade between the eyes without visual loss and eyes with unexplained visual loss following SO removal. We also compared the IOP fluctuations between pre-SO removal and post-SO removal in both group of eyes. The mean IOP fluctuation in eyes with no visual loss following SO removal was 2.4 ± 2 mm Hg during SO tamponade and 1.8 ± 1.2 mm Hg after SO removal (*p* = 0.0001 Wilcoxon matched-pairs signed ranks test). Similarly, the mean IOP fluctuation in eyes who experienced un-explained visual loss following SO removal was 3.1 ± 1.8 mm Hg during SO tamponade and 3.2 ± 1.5 mm Hg after SO removal (p = 0.8203 Wilcoxon matched-pairs signed ranks test). There was no statistically significant difference between the IOP fluctuations following SO removal in eyes with no visual loss after SO removal and eyes with un-explained visual loss (*p* = 0.2114 Wilcoxon matched-pairs signed ranks test). There was also no statistically significant difference in IOP fluctuations during SO tamponade between eyes without visual loss following SO removal and eyes with un-explained visual loss (*p* = 0.0746).

The mean length of time that the eye was filled with SO was 14.8 ± 4.4 months (range 10–24). In comparison, eyes that did not experience visual loss had a mean IOP of 14 ± 7.3 mm Hg (range 10–24; *p* = 0.0002) and a mean tamponade duration of 9.3 ± 10.9 months (range 4–79; *p* < 0.0001). Table 1 summarizes these comparisons.

**Table 1:** Comparison of eyes that suffered and did not suffer an un-explained loss of visual acuity following silicone oil removal

	Un-explained visual acuity loss	No visual acuity loss	*p* value	Odds ratio	95% CI
Age	49.2 ± 16.4	47.2 ± 20.9	0.96		
Gender	11 M 8 F	163 M 98 F	0.81	0.83	0.32–2.13
Cardiovascular disease	0% (0/19)	4.2% (11/260)	1.00	0.90	0.32–2.49
Diabetes mellitus	47.4% (9/19)	29.9% (78/261)	0.13	2.11	0.82–5.40
Systemic hypertension	42.1% (8/19)	28.1% (73/260)	0.20	1.86	0.72–4.82
Giant retinal tear	10.5% (2/19)	6% (17/282)	0.626	1.70	0.39–8.60
Diabetic TRD	47.4% (9/19)	25.5% (72/282)	0.071	2.39	0.93–6.11
PVR	31.6% (6/19)	59.6% (168/282)	0.007	0.261	0.10–0.71
Macula off RD	63.2% (12/19)	66.4% (162/244)	0.80	0.87	0.33–2.29
5000 cs SO	57.9% (11/19)	57.1% (140/245)	0.807	0.84	0.33–2.18
1000 cs SO	42.1% (8/19)	42.9% (105/245)	0.813	0.84	0.32–2.16
BCVA pre SO removal (Snellen)	logMAR = 1.55 ± 0.74 (20/710)	logMAR = 1.43 ± 0.72 (20/538)	0.44		
Baseline pseudophakia	33.3% (6/18)	31% (77/248)	0.80	0.23	0.07–0.75
IOP pre-removal of SO (mm Hg)	19.6 ± 6.9 mm Hg	14 ± 7.3 mm Hg	0.0002		
Duration of SO tamponade (months)	14.8 ± 4.4 months	9.3 ± 10.9 months	<0.0001		

*M* male, *F* female, *TRD* tractional retinal detachment, *PVR* proliferative vitreoretinopathy, *RD* retinal detachment, *cs* centistokes, *SO* silicone oil, *IOP* intraocular pressure

## Discussion

In our retrospective study of 324 eyes of 324 patients who had silicone oil removed from the eye, 7.1% of eyes developed visual loss secondary to known causes. This compares favorably with other reports in the literature. Franks and Leaver [13] reported that 14% of eyes developed retinal re-detachment following SO removal. Kampik et al. [24] reported even higher rates of visual loss following SO removal. In this series, 25% of eyes with severe PDR developed visual loss and over 50% of eyes with PVR developed retinal re-detachment upon SO removal. A more recent study by Choudhary and colleagues [25] reported a 3.5% re-detachment rate following silicone oil removal.

More recently an un-explained visual loss following removal of SO has been recognized [14, 17–20, 26–29]. The incidence of this phenomenon has been reported to be anywhere from 1–30% [18, 29–31]. In the largest series to date, Moya and collaborators [21] found that 14 of 421 (3.3%) eyes that underwent silicone oil removal developed un-explained visual loss. Scheerlinck et al. [22] reported a 30% incidence of un-explained visual loss during silicone oil tamponade or removal. In our current series, 5.9% of eyes developed an un-explained visual loss following SO removal. In all the cases reported in the literature, the fluorescein angiograms as well as the time domain optical coherence tomographies were within normal limits [14, 17–20, 26–28]. Similarly in our current series, the FA and SD-OCT did not reveal any abnormalities that would explain acute changes in visual acuity. We did not perform any electrophysiological studies or visual field testing in any of our patients. In the literature, these studies suggest optic neuropathy, macular dysfunction and generalized retinal dysfunction as causes of the loss of visual acuity [14, 17, 18, 20, 32].

Some investigators have identified young age and macula-on retinal detachments associated with GRT as possible risk factors for an un-explained visual loss following SO removal [18, 27, 30]. In contrast our current series did not confirm these associations. However the small sample size of GRT in our series may have influenced these findings. We identified intraocular pressure and the length of time of silicone oil tamponade as risk factors associated with un-explained visual loss after SO removal. Similarly Scheerlinck and colleagues [29] identified duration of silicone tamponade as a risk factor for the development of un-explained visual loss following silicone oil removal. Marti and co-workers [33] also reported that the most important risk factor for unexplained visual loss in their series was an increased IOP during SO tamponade.

Several hypotheses have been proposed as possible explanations for this phenomenon. According to several authors [17, 18, 32], neuronal apoptosis may be triggered by sudden changes in the ionic flux across the retina [34]. One of the functions of Müller cells is to buffer the extracellular retinal K^+^ concentration by siphoning the excess K^+^ into the vitreous cavity [35]. Long term intraocular SO tamponade may disrupt the ability of the Müller cells to siphon K^+^ into the vitreous leading to an increasing concentration of K^+^ in the subretinal space [36]. Once the SO is removed, the K^+^ concentration undergoes sudden changes that activate apoptosis through caspase-3 and caspase-9 pathways [34]. However a recent study found that the potassium levels are not increased in retro-oil fluid during silicone oil tamponade rendering this hypothesis unlikely [37].

Others have suggested that phototoxicity may play a role in the visual loss following SO removal [20, 30, 31]. Transmission of high-energy blue light is increased in eyes filled with SO particularly in aphakic eyes [38]. In addition SO has previously been shown to dissolve fat-soluble elements such as lutein and zeaxanthin from the retina [39]. Since the fat-soluble macular pigments, lutein and zeaxanthin, are thought to protect the macula from photo-oxidative damage, dissolution of them would render the macula more susceptible to photo-oxidative damage. Dogramaci et al. [31] used a graphic ray computer tracing program to demonstrate that foveal light exposure is increased at the time of SO removal increasing the risk of phototoxicity. To test this hypothesis, the authors compared the visual outcomes of eyes where the SO was removed under direct illumination to SO removal under blocked illumination. Un-explained visual loss was reported in 4.4% of eyes that underwent SO removal under direct illumination compared to only 1.3% under blocked illumination [31]. In contrast, Newsom et al. [30] don’t believe that phototoxicity is of major significance. They argue that the perioperative photostress is limited by the short duration of the procedure. Furthermore light absorption by the lens and cornea helps to mitigate potential phototoxicity of the SO bubble [40]. And finally they report that there is no difference between phakic and pseudophakic eyes with regards to un-explained visual loss after SO removal [30]. Similarly in our cohort of eyes there was no difference in visual loss among pseudophakic and phakic eyes.

Another hypothesis put forward to explain the visual loss following SO removal involves growth factors [18]. Growth factors play an important role in retina homeostasis. Since it is virtually impossible to completely fill an eye with SO, there is always a retro-oil fluid space present. It appears that fibrogenic growth factors such as transforming growth factor beta and interleukin 6 are concentrated in this retro-oil fluid space [41]. SO removal dilutes the concentration of these growth factors. Newsom et al. [18] suggested that cell survival might be affected by changes in growth factor concentration. Removing the SO, which acts as a physical barrier to these substances, allows more widespread dispersal and possibly damage to the macula as a result of accumulation at this site. Williams et al. [28] refute this hypothesis by stating that the fibrogenic growth factors would most likely lead to perisilicone proliferation rather than visual loss. Furthermore there was no evidence of perisilicone membrane formation in the eyes experiencing un-explained visual loss.

Finally SO retinal toxicity has been debated over the years [42, 43]. Experimental studies have shown that SO injection produces vacuoles in the photoreceptor outer segments, thinning and disappearance of the outer plexiform layer, shortening of the horizontal and bipolar processes and swelling of the nerve fiber layer [44, 45]. Numerous histopathological reports of enucleated eyes filled with SO have shown that SO droplets impregnate the iris, ciliary body, trabecular meshwork, optic nerve and retina causing tissue atrophy [46, 47]. Others have shown the absence of intraretinal SO [43]. SO droplets have been identified within the retina in an eye that underwent internal limiting membrane (ILM) peeling and SO tamponade for a macular hole. Curiously, the SO droplets were only present in the retinal area where the ILM had been peeled [48]. Recent advances in multimodality imaging may shed some light in this problem. Mrejen and colleagues [42] noticed that an eye contained countless SO particles despite having had SO removal 11 years prior. This may explain the visual loss experienced by patients during silicone oil tamponade but does not explain the visual loss that occurs immediately after silicone oil removal.

Despite the limitations of the current study which include its retrospective nature, small number of cases and the use of non-standarized visual acuities, our data reflects, to the best of our knowledge, the second largest case series of un-explained visual loss following SO removal. One of the strengths of the current study is that it compares eyes with unexplained visual loss to those without visual loss. Patients who inexplicably lost vision had a higher intraocular pressure and had the oil for more time inside the eye. Maybe a more detailed prospective study that includes automated perimetry, visual evocated potentials, pERG and mfERG, as well as spectral domain OCT could give us some explanations for the visual loss after silicone oil removal.

In summary the incidence of visual loss after removal of silicone oil is important (13%). In about half of these eyes, the etiology of the visual loss can be identified, but in the remaining half the etiology of this complication remains obscure. A higher intraocular pressure and the length of time that the eye is filled with SO could play an important role. Based on this, we advocate SO removal as soon as it is medically feasible and care must be taken to ensure that the IOP remains in a low normal range.
